# Research on the influence of waterproof layer on lining mechanical performance under early temperature load

**DOI:** 10.1371/journal.pone.0321143

**Published:** 2025-04-11

**Authors:** Mingshe Sun, Zhenxiang Guan, Caixia Guo, Xin Xi, Sen Tian

**Affiliations:** 1 School of Hydraulic and Civil Engineering, Ludong University, Yantai, Shandong, China; 2 China Railway 19th Bureau Group Corporation Limited, Beijing, China; 3 Key Laboratory of Urban Security and Disaster Engineering of Ministry of Education, Beijing University of Technology, Beijing, China; 4 Construction Engineering Affairs Service Center of Laishan, Yantai, Shandong, China; Manipal Academy of Higher Education, INDIA

## Abstract

The installation of waterproof layer in the composite lining will change the tangential constraint conditions on the initial support side of lining, affecting the lining mechanical performance under early temperature loads. Based on a loess double track railway tunnel, the influence of tangential constraints on lining mechanical performance under concrete hydration heat temperature load was studied by numerical simulation. Interactions between surrounding rock, initial support, waterproof layer and lining were taken into consideration in the simulation model. The influences of concrete initial pouring temperature, surface heat dissipation coefficient on thermal stress of lining were analyzed and engineering suggestions for preventing early crack were proposed as well. The results show that the greater tangential constraints imposed by the waterproof layer on lining, the greater lining thermal tensile stress, and the higher the risk of through crack. Lining springing constrained by inverted arch is prone to cracking and should be paid more attention in the construction process. Lining thermal stress is significantly affected by concrete initial pouring temperature, and appropriately reducing the pouring temperature is conducive to prevent the occurrence of early cracks. For waterless tunnels, the waterproof layer may be considered to be omitted, and the tangential constraints on the lining can be reduced by installing a thin polyethylene plastic film. The research results can be useful for the design of the composite lining structure.

## 1. Introduction

**Fig 1:**
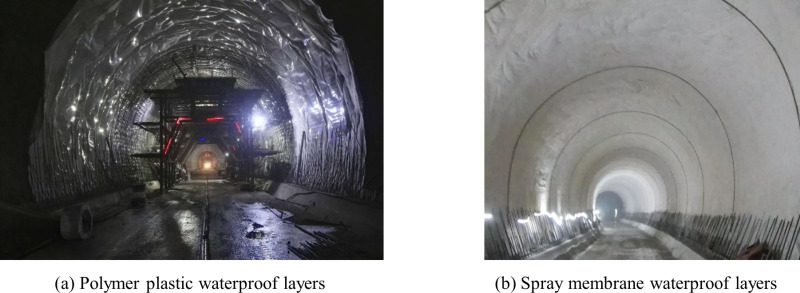
Waterproof layer of composite lining. **(a)** Polymer plastic waterproof layers **(b)** spray membrane waterproof layers.

Tunnels are underground engineering structures, and waterproofing in underground engineering is an important problem. The waterproof layer in composite lining of tunnels plays a crucial role, serving as the main barrier to prevent water leakage in tunnels. In China, the composite lining of tunnels mainly utilizes polymer plastic waterproof layers, which have the advantages of low construction cost and easy operation, as shown in Fig 1a. However, issues such as tearing at the seams of the polymer plastic waterproof layers, cavities between the initial support and the lining, and the disruption of their integrity are common problems [1]. In recent years, spray membrane waterproof layers have gradually been promoted in tunnels, as shown in Fig 1b. This involves using spraying equipment to spray single-component or multi-component waterproof materials onto the structural interface, which coagulates into a membrane in a short time to achieve waterproofing function. The waterproof materials mainly include acrylic salts, polyurea, quick-setting rubber, polyurethane, etc [2,3]. The spray membrane waterproof layer has good interface adaptability and can tightly bond with the initial support and lining, resulting in excellent waterproofing effect. However, this also increases the constraining effect of the initial support on the lining.

Currently, most researches on waterproof layers focus on optimizing its waterproofing effects from the aspects of waterproof materials and construction techniques. Luciani [4] introduced the most widely used technology for suspended waterproofing sheets and elaborated on its key construction points. Qin [5] explored the relationship between pressure loss during Negative Pressure Testing (NPT) and different factors through conducting model experiments and numerical simulations, and proposed the NPT standard for welded scars of waterproofing sheets. Gong et al. [6,7] investigated the seepage and waterproofing issues at the joints of sealing gaskets in shield tunnel linings, exploring the hardening effect of rubber without pre-compression and the stress relaxation behavior of rubber with pre-compression as material characteristics. Yang [8], Holter [9,10], Johnson [11] studied the impermeability, durability, and damage failure characteristics of spray membrane waterproof materials under different groundwater environments, and determined the applicable environments for waterproof materials. Sun [12,13] proposed a novel tunnel waterproof - drainage system based on double - bonded spray membrane materials, and provided the key parameters and applicable scope. Ulrike [14,15] from a practical construction perspective, discussed the advantages and disadvantages of spray membrane waterproof layers, pointing out the questions that need to be answered by all parties involved into the construction process, from the product suppliers to the designers, and the constructor.

However, the installation of the waterproof layer not only plays a structural waterproofing role but also changes the contact characteristics between the initial support and lining, which is also of great significance for ensuring the safety and durability of the lining structure. It is commonly thought that only normal compressive stress is transmitted between the initial support and the lining for the suspended sheet waterproof layer. In contrast, the sprayed membrane waterproof layer can bond the initial support and lining tightly, allowing the interface to transmit normal tensile stress, compressive stress, and shear stress. Regarding the mechanical effect of the waterproof layer in composite lining, Chen [16], Cheng [17] used two-force rod elements, Goodman interface elements, and finite element numerical simulation to study the deformation and mechanical characteristics of the suspended waterproof layer. They found that the composite lining shows a load-bearing characteristic between laminated beams and composite beams. Fang [18] and Wang [19] conducted field tests and numerical simulations to study the mechanical effects of the suspended waterproof layer. Their results indicated that the installation of the suspended waterproof layer affects the cracking of the lining, with continuous cracks appearing in the lining when no waterproof layer is installed. Alan Bloodworth and Jiang Su [20–23] studied the effect of the spray membrane waterproof layer through indoor experiments and numerical simulation. Jiang [24] conducted a four-point bending test on a reinforced concrete beam with a novel polymer waterproofing membrane and calculated the degree of composite action quantitatively. According to their findings, the spray membrane waterproof layer produces a composite effect between initial support and lining. This composite effect enhances the constraint on the lining, making it more susceptible to section cracking. The above research has investigated the mechanical effect of the waterproof layer and beneficial conclusions have been drawn. However, research regarding the influence of waterproof layers on the mechanical performance of the composite lining remains insufficient. Specifically, issues such as the mechanical performance of the lining under temperature loads [25–27] during the construction phase and early temperature induced cracks in the lining have not been fully investigated.

Therefore, this paper comprehensively considers the interactions between the surrounding rock, initial support, waterproof layer, and lining. Using finite element numerical simulation, it studies the influence of the tangential constraint of the waterproof layer on the mechanical behavior of the composite lining structure under the early temperature load. The variations in temperature and stress fields within the lining under both constrained and unconstrained conditions by the initial support were examined. The primary factors influencing the temperature induced stress in the lining were identified, and engineering recommendations for mitigating temperature related cracking in the lining were presented. Finally, the research findings were compared and validated against the field measured cracks in a loess tunnel without a waterproof layer, as documented in reference [18]. The results of this study provide valuable insights for the design of composite lining structures.

## 2. Early mechanical performance of lining structure

**Fig 2:**
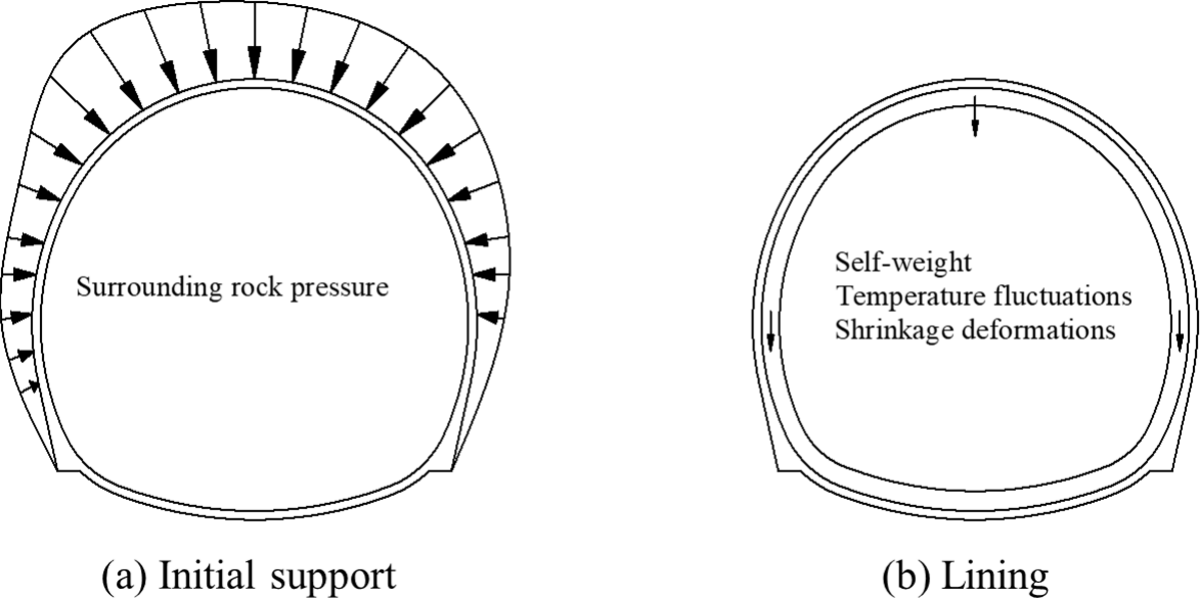
Load action during tunnel construction period. (a) Initial support **(b)** lining.

The loads borne by composite linings depend on the geological and drainage conditions of the tunnel being constructed. For example, linings in hard rock drainage tunnels or loess tunnels are unlikely to bear significant ground pressure and water pressure during their service life. Upon tunnel excavation, the initial support is promptly installed to contain the deformation of the surrounding rock and ensure its stability. The lining is typically constructed after the deformation of the surrounding rock and initial support has stabilized. Hence, the early primary loads governing the lining behavior are expected to be its own self-weight along with those induced by environmental variations, such as temperature fluctuations and shrinkage deformations, as shown in Fig 2.

**Fig 3:**
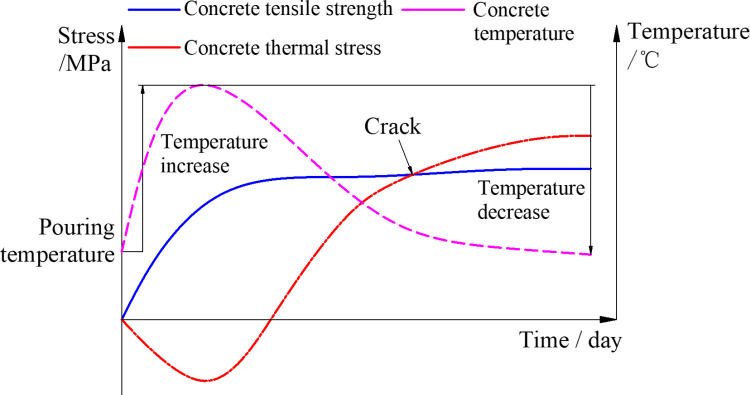
Mechanical mechanism model of lining concrete crack.

In the early stages following the pouring of lining concrete, the lining is susceptible to deformation resulting from the release of hydration heat and shrinkage of concrete. The installation of the waterproof layer will constrain this lining deformation, and a stronger deformation constraint will consequently lead to greater internal stress within the lining. If the internal stress within the lining surpasses the concrete tensile strength, the lining is likely to crack, as shown in Fig 3. Cracks of the lining can easily lead to tunnel leakage, reinforcement corrosion, and spalling of the lining, which significantly affects the operational safety and structural durability of the tunnel. Therefore, studying the impact of waterproof layer constraints on the mechanical performance of lining under the influence of construction temperature loads is of significant engineering importance.

## 3. Numerical simulation calculation

To demonstrate how the waterproof layer’s constraints affect the temperature stress of the lining, the following finite element numerical simulation examines the stress characteristics of the lining when subjected to the heat generated by concrete hydration during the construction period.

### 3.1. Numerical calculation model

**Fig 4:**
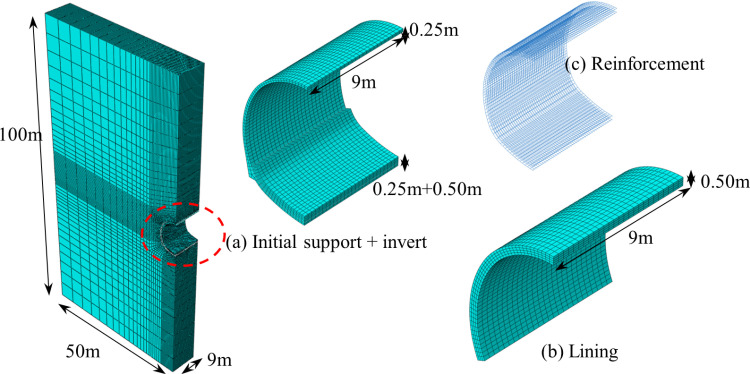
Finite element numerical model.

Considering the combined effects of the self-weight of the lining structure and the heat release from concrete hydration, a finite element numerical calculation model has been established for a double-track railway tunnel constructed in loess strata. The excavation width and height of the tunnel are 11.2 meters and 11.0 meters, respectively. The tunnel initial support consists of shotcrete and steel mesh frames with a thickness of 0.25 m and the lining is made of reinforced concrete with a thickness of 0.50 m. The longitudinal pouring length of the concrete for the tunnel lining is 9 m. The lining is poured starting from the base and progressively proceeds toward the sidewalls and the vault. It is assumed that the lining concrete is cast in a single pour. Taking advantage of the symmetry of the problem, a half-scale model of the overall structure is established as the computational model using ABAQUS finite element software. The model dimensions are 50 m × 100 m × 9 m, as shown in Fig 4.

### 3.2. Numerical calculation conditions

#### 3.2.1. Calculation parameters of concrete and surrounding rock.

**Fig 5:**
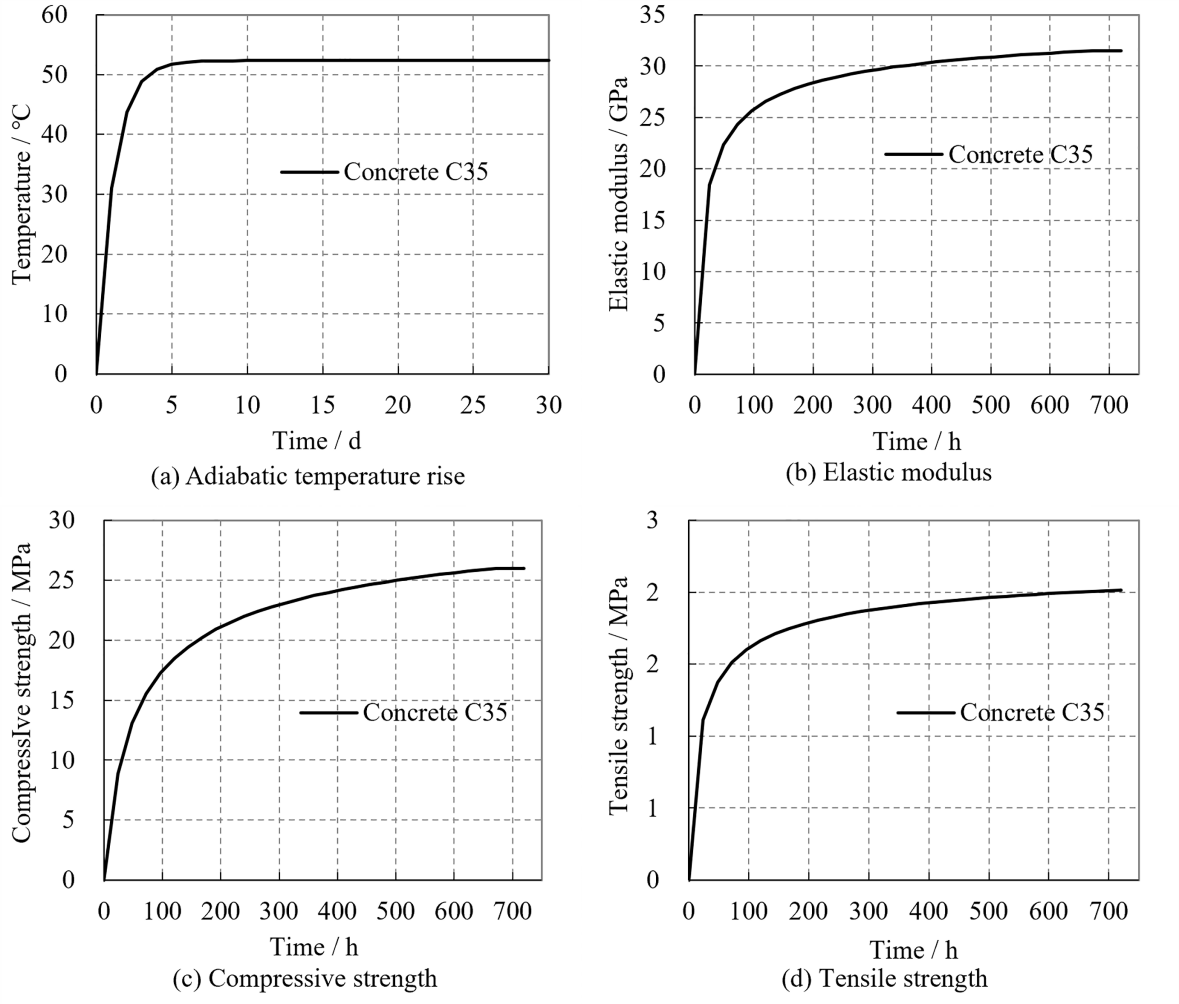
The numerical calculation parameters of concrete. **(a)** Adiabatic temperature rise. **(b)** Elastic modulus. **(c)** Compressive strength. **(d)** Tensile strength.


The initial support of the tunnel utilizes C25 shotcrete with a density of *γ* = 23.5 kN/m³ and the lining is constructed with C35 concrete with a density of *γ* = 24.0 kN/m³. The specific heat capacity of the concrete is c = 0.96 kJ/kg·°C, the thermal conductivity is λ = 10.6 kJ/m·h·°C, and the linear expansion coefficient is *α* = 10^–5^/°C. The hydration exothermic process of concrete is characterized by an exponential curve, as shown in the following equation and Fig 5a:

$$\theta \left(t\right)=\theta_{0}\left(1-e^{-m\;t}\right)$$ (1)

where *θ*(*t*) represents the adiabatic temperature rise of the concrete; *θ*₀ is the final value of the adiabatic temperature rise; *t* is the age of concrete; and *m* is a constant. In this calculation model, the final adiabatic temperature rise *θ*₀ is 52.33 °C, and the constant *m* is 0.9. Considering that the elastic modulus and strength of concrete increase with age, the elastic modulus, compressive strength, and tensile strength of the concrete are determined according to equations (2), (3), and (4) respectively [28,29].


$$E_{\text{c}}\left(t\right)=E_{\text{c}}\sqrt{e^{s\left(1-\sqrt{28/t}\right)}}$$ (2)

$$f_{\text{c}}\left(t\right)=e^{s\left(1-\sqrt{28/t}\right)}\cdot f_{\text{c}}$$ (3)

$$f_{\text{t}}\left(t\right)=0.88k\cdot \left(0.395f_{\text{c}}^{0.55}\left(t\right)\right)\cdot {\left(1-1.645\delta \right)}^{0.45}$$ (4)

where *E*_c_(t) represents the elastic modulus of the concrete at age *t* (in days) in GPa; *E*_c_ is the elastic modulus of the concrete at 28 days; *f*_c_(t) is the compressive strength of the concrete at age *t* in MPa; *f*_c_ is the compressive strength of the concrete at 28 days; *f*_t_(t) is the tensile strength of the concrete at age t (in days) in MPa; *s* is a coefficient related to the type of cement, with s = 0.25 for both ordinary and rapid hardening cements; *k* is the brittleness coefficient of concrete; *δ* is the coefficient of variation for the compressive strength of cubes. The variation curves of these parameters with concrete pouring time are shown in Fig 5b–5d.

According to the engineering geological survey report for the tunnel, the strata are predominantly composed of upper pleistocene aeolian sandy loess and clayey loess, as well as middle pleistocene alluvial sandy loess and clayey loess. The main physical and mechanical parameters of the loess are as follows: density *γ* is 17.0kN/m^3^, elastic modulus *E*_loess_ is 500 MPa, Poisson’s ratio *μ* is 0.35; specific heat capacity *c* is 2.5kJ/kg·°C, and thermal conductivity *λ* is 4.3kJ/m·h·°C [30].

#### 3.2.2. Initial and boundary conditions.

**Fig 6:**
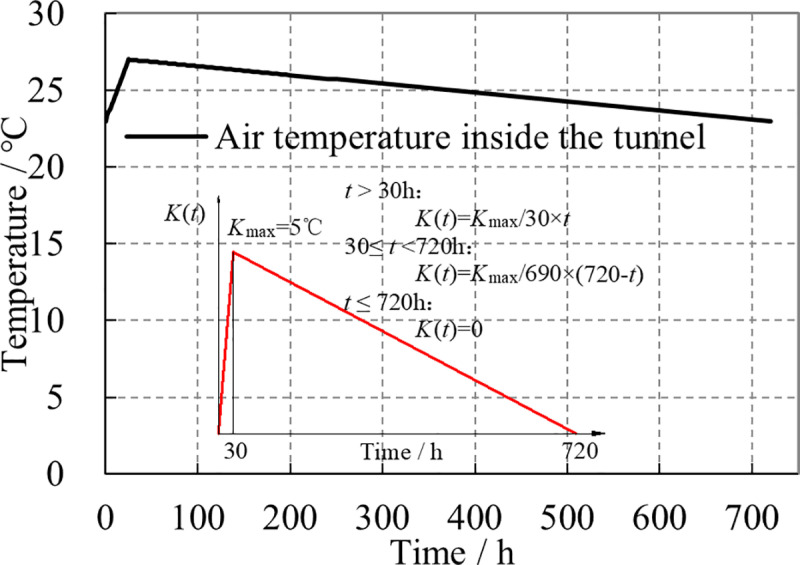
Air temperature in tunnel with lining construction time.

Typically, the variation in strata temperature is small and relatively uniform. The temperature in the strata near the ground surface is generally close to the monthly average of air temperatures, and the temperature in the strata below 10 meters from the ground surface is essentially the same as the annual average of air temperatures. Based on the annual average air temperature of the tunnel site area, the temperature of the surrounding rock (loess) is determined as 18 °C. Before the construction of lining, the initial support and the inverted arch have been completed. Thus, their temperatures can be assumed to be the same as that of the surrounding rock, which is 18 °C. The lining construction is carried out from September to October, with the pouring temperature of the lining concrete set at 28 °C. Concurrently, the initial air temperature inside the tunnel is recorded at 23 °C. Furthermore, considering the enclosed nature of the tunnel interior, the heat generated by machinery during the lining concrete pouring process, along with the heat of hydration from the concrete itself, may lead to an air temperature increase inside the tunnel. Therefore, the air temperature inside the tunnel is considered to vary with the construction time of the lining [31], as shown in Fig 6. The red curve represents the variation curve of the air temperature difference inside the tunnel, where *K*(*t*) denotes the temperature difference between the tunnel’s internal air temperature and the initial temperature.

**Fig 7:**
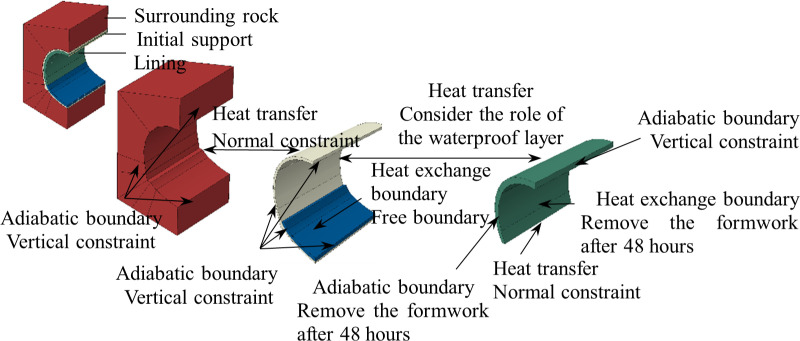
Boundary conditions of finite element numerical model.

The model boundary conditions include thermal boundaries and mechanical boundaries, as shown in Fig 7. The thermal boundaries are defined by the surrounding rock, initial support and lining. The surfaces of surrounding rock, the front and back surfaces of both the initial support and lining, as well as the symmetrical surface of the model, are all thermally insulated. Heat exchange can occur between the lining concrete and the air within the tunnel. Additionally, the surrounding rock and the initial support, as well as the initial support and the lining, can conduct heat transfer. The heat dissipation coefficient on the inner surface of the lining concrete is influenced by the airflow speed within the tunnel. However, considering the limited ventilation capacity and low wind speeds within the tunnel, and neglecting the insulating effect of the formwork, the heat dissipation coefficient for the lining surface is taken as 36 kJ/m²·h·°C.

The mechanical boundary mainly consists of displacement constraints for each part. The vertical displacement of the surrounding rock on all lateral and symmetrical surfaces is fixed. The vertical displacement of the front and back surfaces, as well as the symmetrical surface, of both the initial support and the lining is fixed. Additionally, the displacement of the lining’s inner surface is also fixed due to the constraints imposed by the formwork.

In summary, the thermal and mechanical parameters for the finite element numerical calculations are presented in Table 1.

**Table 1:** The thermal and mechanical parameters for the finite element numerical model.

Program		Lining	Inverted arch	Initial support	Surrounding rock
Parameters	Unit	C35	C35	C25 shotcrete	Loess
Initial temperature	°C	28	18	18	18
Specific heat capacity*c*	kJ/ kg·°C	0.96	0.96	0.96	2.5
Thermal conductivity*λ*	kJ/ m·h·°C	10.6	10.6	10.6	4.3
Coefficient of linear expansion*α*	/°C	10^-5^	10^-5^	10^-5^	–
Density*γ*	kN/ m^3^	24.0	24.0	23.5	17.0
Elastic modulus	GPa	*E*_c_(*t*)	31.5	23	0.5
Poisson’s ratio *μ*	–	0.2	0.2	0.2	0.35

## 4. Numerical calculation results and analysis

### 4.1. Temperature field calculation results and analysis

**Fig 8:**
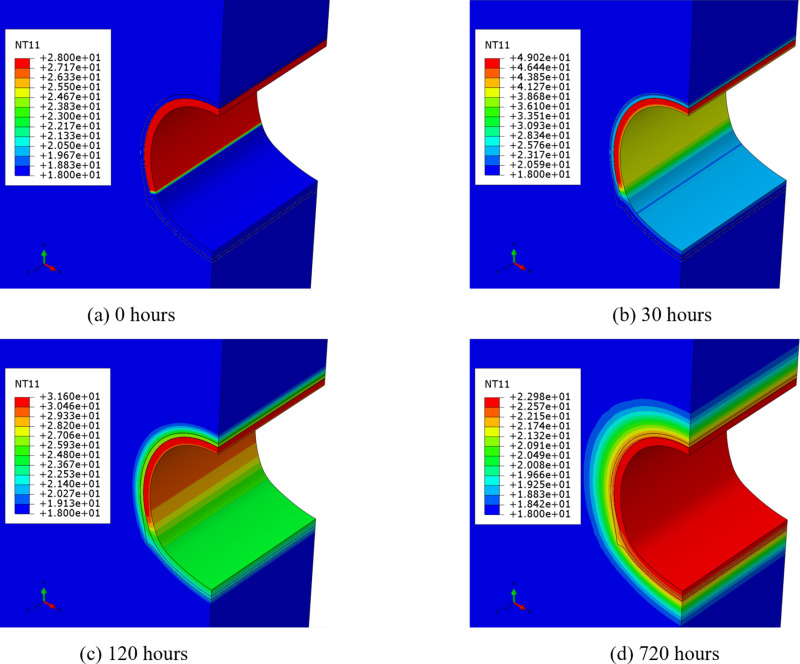
Temperature field at different time after lining concrete pouring. **(a)** 0 hours **(b)** 30 hours **(c)** 120 hours **(d)** 720 hours.

Using the above finite element simulation model and parameters, the construction process of the lining and the hydration heat release process of concrete were simulated. Consequently, the temperature fields of surrounding rock, initial support and lining at different moments were obtained. Owing to the thinness of the waterproof layer, its insulating effect was not considered in the simulation, and it was assumed that the temperature and heat flux at the interface between the initial support and the lining are continuous. Therefore, although the mechanical constraints are different, the temperature field results for the composite lining waterproof layer are essentially the same. The distribution results of the temperature field after 0 hours, 30 hours, 120 hours, and 720 hours of lining concrete pouring are shown in Fig 8.

It can be observed that as the concrete pouring time increases, the temperatures of the initial support, inverted arch, and surrounding rock gradually rise, and the hydration heat of the concrete is transmitted to a certain range before it begins to stabilize.

In the early stage of concrete pouring, the temperature of the lining increases rapidly, reaching the highest temperature of 49.0 °C after 30 hours, with a temperature increase of 21.0 °C. As a result of heat transfer, the temperature of the initial support is now approximately 25.0 °C, with a temperature increase of about 7.0 °C. Currently, the temperature of the surrounding rock is not significantly affected by heat transfer.

After 120 hours of concrete pouring, the highest temperature of the lining decreases to 31.6 °C, while the temperature of the initial support remains essentially stable at approximately 25.0 °C. The surrounding rock temperature is about 23.0 °C, with a temperature increase of about 5.0 °C, and the depth affected by hydration heat is about 0.5 m.

After 720 hours of concrete pouring, the temperature distribution of the lining, initial support, inverted arch, and surrounding rock becomes uniform and equal to the air temperature inside the tunnel, with a temperature value of 23 °C. The depth of the surrounding rock affected by the concrete hydration heat is about 1.5 to 2.0 m.

**Fig 9:**
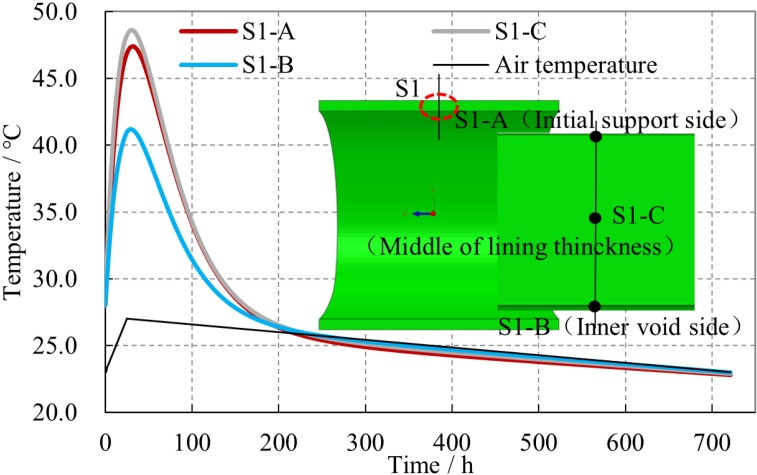
Temperature curves at lining vault with concrete construction time.

Considering that the vault position of the lining is relatively important, the crown section of the lining is selected to observe the variation of its temperature. The temperature variation curves of different observation points on the initial support side (S1-A), inner void side (S1-B), and the middle of lining thickness (S1-C) with time were obtained, as shown in Fig 9.

It can be observed that along the thickness direction of the lining, the temperature variation characteristics of each observation point are basically the same, showing a trend of rapidly increasing to their peak values and then gradually decreasing to a stable state. About 30 hours after the lining concrete pouring, the temperature of each observation point reached its respective peak value, with the highest temperature being 49.0 °C, located at the center of the lining thickness (S1-C). Subsequently, 200 hours after the concrete pouring, the temperature of each observation point fell to levels roughly consistent with the air temperature inside the tunnel. Influenced by the initial support and the air temperature inside the tunnel, the highest temperature within the lining section is located at the center of the thickness. The temperature near the initial support surface is lower than that at the center, and the temperature near the inner void surface is the lowest. The maximum temperature difference between the inner and outer surfaces at each observation point in the lining section is approximately 7.5 °C.

### 4.2. Stress field calculation results and analysis

**Fig 10:**
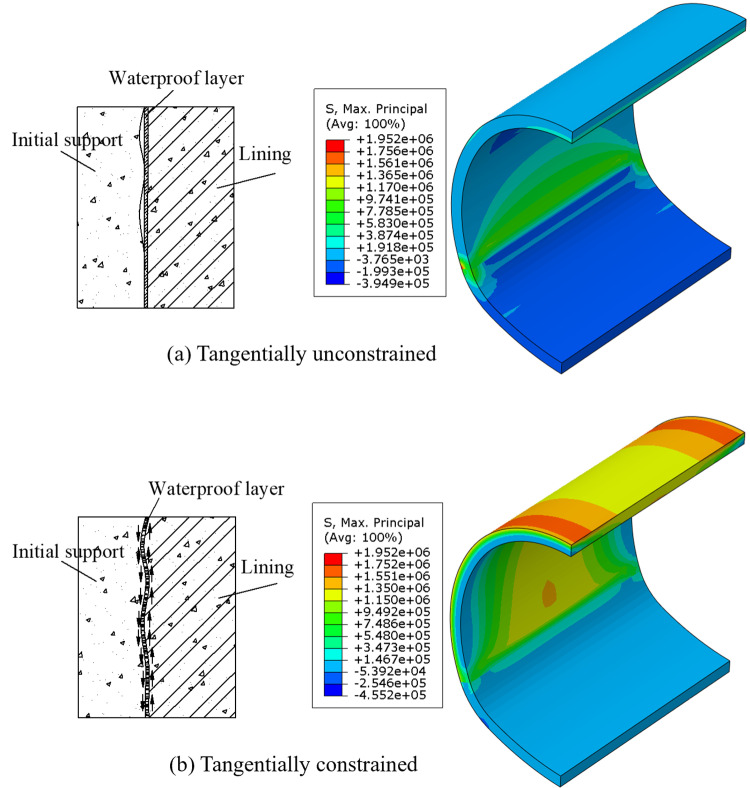
The maximum principal stress of lining. **(a)** Tangentially unconstrained. **(b)** Tangentially constrained.

Considering the tangential constraint effect of the waterproof layer, the sequential coupling thermal stress analysis method [32,33] was utilized to evaluate the temperature stress of the lining under different conditions. The constraint effect of the waterproof layer is divided into two scenarios, with and without tangential constraint. The construction process of the lining is cast entirely in a single pour, with the formwork constraint removed 48 hours after concrete pouring. So, the lining is subjected to its own weight and the temperature loads. After 720 hours of concrete pouring, the distributions of the maximum principal stress of the lining under the two different conditions are shown in Fig 10.

It can be observed that when the lining is tangentially unconstrained by the waterproof layer, tensile stresses at the inner side of the crown and springing are relatively high, with stress values ranging from 0.58 to 0.78 MPa. However, when the lining is tangentially constrained by the waterproof layer, the entire lining experiences tensile stresses that are relatively high. The maximum stress is observed in the regions 2.5 m from both ends along longitudinal direction of the lining, adjacent to the initial support, reaching a peak value of 1.95 MPa. In the mid-span of the lining, almost the entire cross section is under tension, with tensile stress values ranging from 1.15 to 1.35 MPa. This indicates that the greater the constraint provided by the waterproof layer, the more likely it is to induce larger temperature-induced tensile stresses within the lining, which could lead to the occurrence of early cracks.

**Fig 11:**
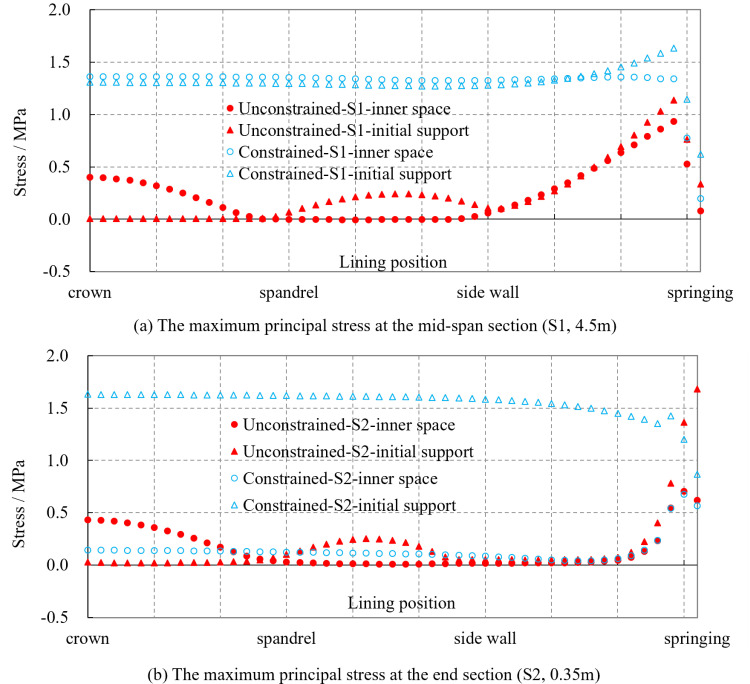
The maximum principal stresses of middle and end section of lining. **(a)** The maximum principal stress at the mid-span section (S1, 4.5 m). **(b)** The maximum principal stress at the end section (S2, 0.35 m).

To illustrate the mechanical characteristics of the lining, two sections at the mid-span (S1, 4.5 m) and the end (S2, 0.35 m) of the lining are selected. The distributions of the maximum principal stress along the circumferential direction of the tunnel at these two sections are shown in Fig 11 after 720 hours of lining pouring.

In the case of the mid-span section (S1, 4.5 m), it is observed that when the lining is tangentially constrained by the waterproof layer, the tensile stresses on both the inner void side and the initial support side of the lining, from the crown to the springing, are relatively close, ranging approximately from 1.3 to 1.4 MPa. This indicates that the section is in a state of pure tensile stress. In contrast, when the lining is tangentially unconstrained, the tensile stress on the inner void side from the crown to the spandrel is higher than that on the initial support side, whereas the opposite trend is observed from the spandrel to the sidewall, with stress values ranging from 0.2 to 0.4 MPa. This suggests that the lining is subjected to bending moments. From the sidewall to the springing, the stress values on both sides are similar and gradually increase to 1.0 MPa. Furthermore, from the crown to the sidewall, the tensile stress in the lining section under tangential constraint is significantly higher than that without constraint, with an increase of 1.0 to 1.4 MPa. Under both conditions, the tensile stress at the springing region of the lining remains relatively high, with stress values being close, approximately 1.1 to 1.3 MPa.

As for the end section (S2, 0.35 m), observations indicate that when the lining is tangentially constrained by the waterproof layer, the tensile stress on the initial support side of the lining from the crown to the springing is significantly greater than that on the inner void side, with a stress difference of about 1.5 MPa. This suggests significant bending moments are present in the lining. Similarly, when the lining is tangentially unconstrained, the tensile stress from the crown to the springing is essentially the same as that at the mid-span section, with the maximum tensile stress occurring at the lining springing.

Thus, the internal forces at both the mid-span section and the end section of the lining are similar when it is tangentially unconstrained by the waterproof layer, indicating that the lining is under a uniform load. The peak tensile stress is located at the springing. On the contrary, when the lining is tangentially constrained by the waterproof layer, there is a noticeable difference in internal forces between the mid-span and end sections of the lining. The lining is under pure tensile stress with a relatively high value of about 1.3 MPa at the mid-span section. Whereas, the lining is subjected to significant bending moments at the end section, with a higher tensile stress of about 1.6 MPa on the initial support side of the lining.

In summary, under the loads of temperature and self-weight, the different tangential constraints imposed by waterproof layer can result in different stress states within the lining during its construction period. The presence of tangential constraint from the waterproof layer significantly increases the tensile stress in the mid-span section of the lining, with an increase of approximately 1.0 to 1.4 MPa. The stronger the tangential constraint, the greater the tensile stress, thereby leading to a higher likelihood of through-thickness cracking in the lining. Meanwhile, the lining will be subjected to higher bending moments at the end section. Additionally, the tensile stress at the lining springing is relatively high, making it prone to cracking. More attention should be paid during the lining construction process. This phenomenon occurs because linings are typically made of concrete, a material known for its high compressive strength but low tensile strength, making it prone to cracking under significant tensile stress. At the springing region, the geometric shape and stress characteristics make it particularly susceptible to stress concentration. Additionally, this area often interacts with other structural elements, such as the invert, which is constructed earlier. The invert constrains the thermal expansion and contraction of the concrete in this region, thereby increasing the thermal tensile stress and leading to cracking. Furthermore, factors such as inadequate vibration during construction, insufficient curing, groundwater infiltration, and surrounding rock pressure can further exacerbate the likelihood of cracking at the springing region.

**Fig 12:**
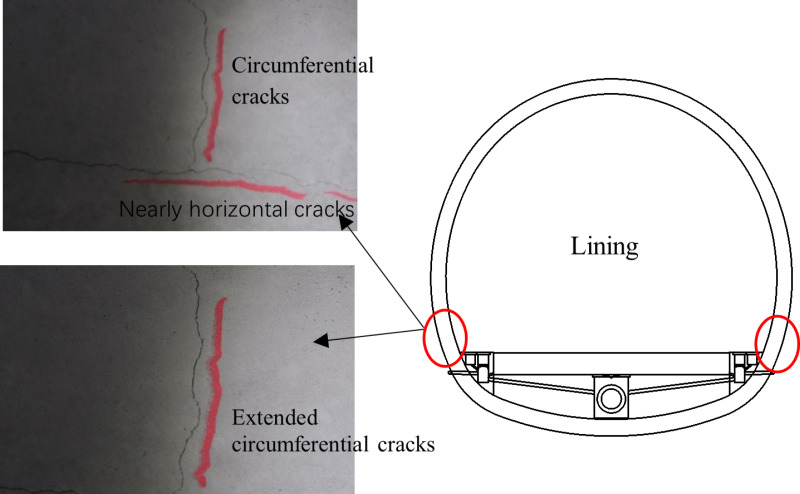
Lining circumferential crack and nearing horizontal crack [ 18].

A field experiment was conducted in a loess tunnel as described in reference [18], where the waterproof layer between the initial support and lining was removed. This action led to significant tangential constraints being imposed on the lining. The circumferential cracks in the arch part are generally distributed above the nearly horizontal cracks, with lengths ranging from 1.5 to 4.5 meters, and mainly occur in the middle part of the lining. These cracks are shown in Fig 12. The monitoring results of the field experiment are consistent with the theoretical results presented in this paper. Therefore, for the placement of the waterproof layer between the initial support and lining, while optimizing construction techniques and waterproofing effectiveness, efforts should be made to reduce its tangential constraints on the lining to prevent early cracks.

## 5. Factors affecting thermal stress and engineering recommendations

### 5.1. Factors affecting thermal stress

The aforementioned analysis and discussion suggest that during lining construction period under the influence of temperature and self-weight loads, the greater the tangential constraints imposed by the waterproof layer on the lining, the more likely it is to result in significant thermal tensile stress, leading to early cracking of the lining. Below is the analysis of factors affecting the thermal stress in the lining. It primarily considers the impact of the initial pouring temperature of lining concrete and its surface heat dissipation coefficient. The initial pouring temperatures of lining concrete are taken as 23 °C, 28 °C, and 33 °C, and the surface heat dissipation coefficients are 24 kJ/m²·h·°C, 36 kJ/m²·h·°C, and 54 kJ/m²·h·°C. The finite element numerical simulation model and parameters remain the same as before. The scenario in which the lining at the initial support side is constrained by the waterproof layer was selected for research and analysis. The mid-thickness point of the crown section at the mid-span (S1-C) is taken as the monitoring point to analyze the characteristics of the circumferential stress *σ*_22_ and the axial stress *σ*_33_.

#### 5.1.1. Impact of concrete initial pouring temperature.

**Fig 13:**
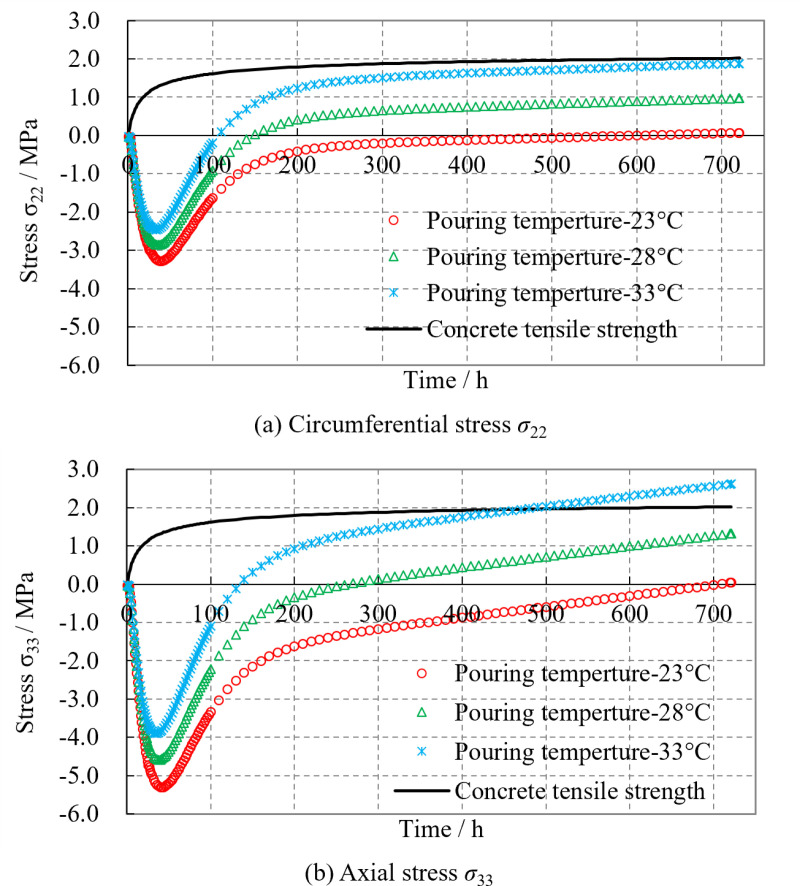
The influence of concrete initial pouring temperature on lining stress. **(a)** Circumferential stress *σ*_22_. **(b)** Axial stress *σ*_33_.

The concrete initial pouring temperature is an important factor affecting the thermal stress of the lining. Fig 13 shows the time variation curves of lining thermal stresses under different initial pouring temperatures of concrete

It can be observed that although the initial pouring temperatures of concrete are different, the variation characteristics of the lining thermal stress with time are essentially consistent. For every 5 °C increase in the initial pouring temperature of concrete, the circumferential and axial thermal stresses of the lining increase by approximately 1.0 MPa. However, when the initial pouring temperature is 33 °C, the circumferential stress of the lining approaches the concrete ultimate tensile strength, and the axial stress has exceeded its tensile strength, resulting in cracking of the lining. The results suggest that the concrete initial pouring temperature significantly affects the thermal stress of the lining, and reducing the concrete pouring temperature appropriately is beneficial in preventing the occurrence of early cracks in the lining. Therefore, it is recommended that the temperature difference between the concrete pouring temperature and the tunnel air temperature be kept within 5 °C during tunnel lining construction, and it should not exceed 10 °C. Otherwise, it may lead to cracking of the lining.

#### 5.1.2. Impact of the surface heat dissipation coefficient of concrete.

**Fig 14:**
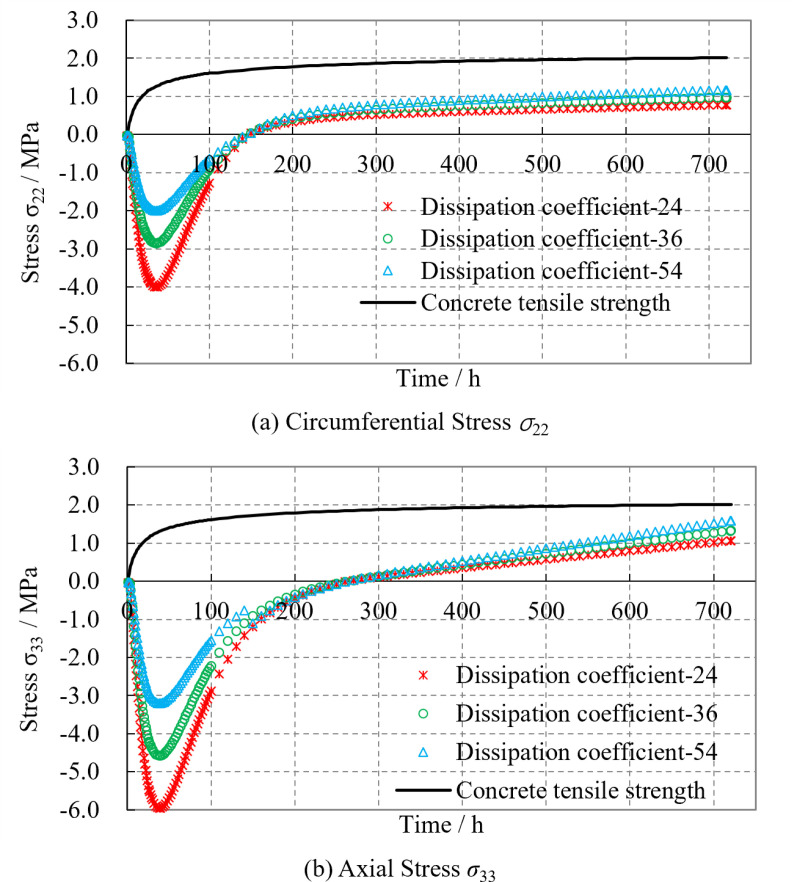
The influence of concrete heat dissipation coefficient on lining stress. **(a)** Circumferential Stress σ_22_. **(b)** Axial Stress σ_33_.

The magnitude of the concrete surface heat dissipation coefficient is influenced by the wind speed inside the tunnel. Specifically, the greater the wind speed, the greater the heat dissipation coefficient on the concrete surface. Fig 14 illustrates the time variation curves of the lining thermal stresses under different concrete surface heat dissipation coefficients.

It can be observed that regardless of the different surface heat dissipation coefficients of concrete, the variation characteristics of the lining thermal stresses with time are essentially consistent. The circumferential and axial thermal stresses of the lining increase rapidly from zero to their maximum values, then gradually decrease, and finally transition to tensile stress. As the concrete surface heat dissipation coefficient increases from 24 to 54 kJ/m²·h·°C, for every 50% increment in the coefficient, the maximum thermal compressive stress of the lining decreases by 20% to 30% (the circumferential stress decreases from -4.0 to -2.0 MPa, and the axial stress decreases from −6.0 to −3.2 MPa). Simultaneously, the thermal tensile stress of the lining increases by 20% to 25% (the circumferential stress increases from 0.8 to 1.2 MPa, and the axial stress increases from 1.1 to 1.6 MPa). The greater the surface heat dissipation coefficient, the smaller the peak value of the circumferential and axial thermal compressive stresses of the lining, and the greater the final tensile stresses. This is because a higher surface heat dissipation coefficient of concrete indicates a faster rate of heat exchange between the lining concrete and the surrounding air inside the tunnel. As a result, the hydration heat of the lining concrete is transferred more rapidly to the surrounding environment, leading to a relatively smaller temperature increase during the heating phase. Consequently, the expansion deformation of the concrete is reduced, resulting in lower thermal compressive stresses. During the cooling phase, the concrete temperature decreases more rapidly, causing more significant shrinkage and thereby generating higher thermal tensile stresses. The final circumferential stress is approximately 1.2 MPa, and the axial stress is approximately 1.6 MPa, approaching the tensile strength of the concrete. Compared with the concrete initial pouring temperature, the surface heat dissipation coefficient has a smaller impact on the thermal stress of the lining. However, it is also important to maintain a stable wind speed inside the tunnel during lining construction, especially at the tunnel entrance.

### 5.2. Engineering recommendations

**Fig 15:**
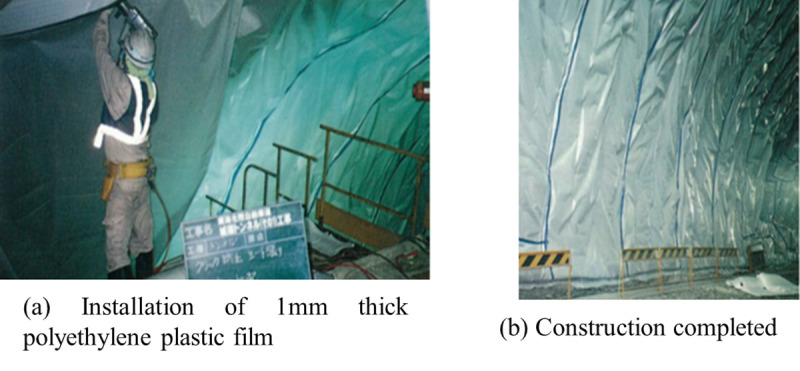
Installation of polyethylene plastic film.

Through the previous discussion, it can be concluded that the tangential constraint effect of the waterproof layer has a significant impact on the stress performance of the lining and the occurrence of early cracks. For waterless tunnels, such as loess tunnels, the role of the waterproof layer cannot be effectively utilized, and its installation could be considered to be omitted. Consequently, the lining will be subjected to significant tangential constraints. Therefore, to reduce the tangential constraint impact on the lining, a thin polyethylene plastic film can be installed between the initial support and the lining, as shown in Fig 15. This measure can prevent the occurrence of early thermal cracks in the lining.

## 6. Conclusions

In this paper, the impact of the tangential constraints imposed by the waterproof layer on the mechanical performance of the lining was investigated. The study took into comprehensive consideration the interactions among the surrounding rock, initial support, waterproof layer, and lining, and established a finite element numerical simulation model. Utilizing the numerical model, the characteristics of lining thermal stress variation and the influencing factors were analyzed after the lining concrete pouring. Ultimately, recommendations for the prevention of lining thermal cracks and the placement of the waterproof layer were obtained. The main conclusions are as follows:

1. The presence of tangential constraint from the waterproof layer significantly increases the tensile stress in the mid-span section of the lining, with an increase of approximately 1.0 to 1.4 MPa. The stronger the tangential constraint, the greater the tensile stress, thereby leading to a higher likelihood of through-thickness cracking in the lining. Meanwhile, the lining will be subjected to higher bending moments at the end section. The thermal tensile stress at the lining springing is relatively high, making it prone to cracking. More attention should be paid to these positions during the lining construction process.
2. The influence of concrete initial pouring temperature on the thermal stress of the lining is significant. Reducing the initial pouring temperature appropriately is beneficial in preventing the occurrence of lining early cracks. The temperature difference between the concrete pouring temperature and the tunnel air temperature should ideally be kept within 5 °C. The larger the surface heat dissipation coefficient of the concrete, the smaller the early thermal compressive stress of the lining, and the greater the induced circumferential and axial tensile stresses.
3. For waterless tunnels, the waterproof layer may be considered to be omitted. Instead, a thin polyethylene plastic film can be installed between the initial support and the lining to reduce the tangential constraint impact on the lining, thereby preventing the occurrence of early thermal cracks.

This review discusses the impact of the waterproof layer on the mechanical performance of lining under the early temperature loads through numerical simulation methods and proposes engineering suggestions for preventing early thermal cracks in linings. However, in the numerical simulation, the lining was assumed to be cast in a single pour, neglecting the construction sequence and process of the lining concrete. This assumption introduces a certain level of discrepancy compared to actual engineering practices. Additionally, further field tests in tunnel engineering or indoor model tests should be conducted to verify the applicability of these suggestions.

## Supporting information

S1 Data Source data for Figs 5, 9, 11–14.
(XLSX)
